# Recommendations for Lung Ultrasound in Internal Medicine

**DOI:** 10.3390/diagnostics10080597

**Published:** 2020-08-16

**Authors:** Natalia Buda, Wojciech Kosiak, Marcin Wełnicki, Agnieszka Skoczylas, Robert Olszewski, Jakub Piotrkowski, Szymon Skoczyński, Elżbieta Radzikowska, Ewa Jassem, Elżbieta Magdalena Grabczak, Piotr Kwaśniewicz, Gebhard Mathis, Tudor P. Toma

**Affiliations:** 1 Department of Internal Medicine, Connective Tissue Diseases and Geriatrics, Medical University of Gdansk, 80-365 Gdansk, Poland; 2 Department of Pediatrics, Hematology and Oncology, Medical University of Gdansk, 80-365 Gdansk, Poland; kwojtek@gumed.edu.pl; 3 3rd Department of Internal Medicine and Cardiology, Medical University of Warsaw, 02-097 Warsaw, Poland; welnicki.marcin@gmail.com; 4 Geriatrics Department, National Institute of Geriatrics, Rheumatology and Rehabilitation, 02-637 Warsaw, Poland; agnieszka.skoczylas@spartanska.pl; 5 Department of Gerontology, Public Health and Didactics, National Institute of Geriatrics, Rheumatology and Rehabilitation, 02-637 Warsaw, Poland; robert.olszewski@me.com; 6 Department of Ultrasound, Institute of Fundamental Technological Research, Polish Academy of Sciences, 02-106 Warsaw, Poland; 7 Department of Internal Medicine and Gastroenterology, Independent Public Health Care Facility of the Ministry of the Internal Affairs with the Oncology in Olsztyn, 10-900 Olsztyn, Poland; kuba.piotrkowski@gmail.com; 8 Department of Pneumonology, Faculty of Medical Sciences in Katowice, Medical University of Silesia, 40-055 Katowice, Poland; simon.mds@poczta.fm; 9 III Department of Lung Diseases and Oncology, National Tuberculosis and Lung Diseases Research Institute, 01-138 Warsaw, Poland; e.radzikowska@wp.pl; 10 Department of Pulmonology and Allergology, Medical University of Gdansk, 80-210 Gdansk, Poland; ejassem@gumed.edu.pl; 11 Department of Internal Medicine, Pulmonary Diseases and Allergy, Medical University of Warsaw, 02-097 Warsaw, Poland; mgrabczak@vp.pl; 12 Diagnostic Imaging Department, Mother and Child Institute, 01-211 Warsaw, Poland; kwasniewiczp@gmail.com; 13 Emergency Ultrasound in the Austrian Society for Ultrasound in Medicine and Biology, 1100 Vienna, Austria; gebhard.mathis@cable.vol.at; 14 Consultant Respiratory Physician and Honorary Clinical Senior Lecturer, King’s College University Hospital Lewisham and Greenwich NHS Trust, London SE6 2LR, UK; ttoma@doctors.org.uk

**Keywords:** lung ultrasound, chest ultrasound, internal medicine, recommendations

## Abstract

A growing amount of evidence prompts us to update the first version of recommendations for lung ultrasound in internal medicine (POLLUS-IM) that was published in 2018. The recommendations were established in several stages, consisting of: literature review, assessment of literature data quality (with the application of QUADAS, QUADAS-2 and GRADE criteria) and expert evaluation carried out consistently with the modified Delphi method (three rounds of on-line discussions, followed by a secret ballot by the panel of experts after each completed discussion). Publications to be analyzed were selected from the following databases: Pubmed, Medline, OVID, and Embase. New reports published as of October 2019 were added to the existing POLLUS-IM database used for the original publication of 2018. Altogether, 528 publications were systematically reviewed, including 253 new reports published between September 2017 and October 2019. The new recommendations concern the following conditions and issues: pneumonia, heart failure, monitoring dialyzed patients’ hydration status, assessment of pleural effusion, pulmonary embolism and diaphragm function assessment. POLLUS-IM 2020 recommendations were established primarily for clinicians who utilize lung ultrasound in their everyday clinical work.

## 1. Introduction

The progress that has occurred since the publication of the first recommendations for the use of lung ultrasound in internal medicine POLLUS-IM 2018 [1] prompts their update. The last systematic review of relevant literature was completed in August 2017, and 275 original papers were further analyzed. Between August 2017 and October 2019, 253 new original papers devoted to the use of lung ultrasound in adult patients not treated in Intensive Care Units were published. The need to update the recommendations correlates with the rapidly enlarging group of physicians who perform lung ultrasound (LUS) exams in their everyday clinical practice. The working group involved in the updating of the recommendations is a multidisciplinary team, consisting of 11 specialists in internal medicine, pulmonology, cardiology, geriatrics, radiology and pediatrics. Two specialists in lung ultrasound who supervised the updating process (GM and TT) were also involved. The working group was established based on participation application form and experience in lung ultrasound.

## 2. Materials and Methods

The process of formulating the recommendations consisted of the following successive stages: (a) literature review and selection, (b) creation of a database, (c) formulation of statements, (d) literature data credibility analysis, (e) discussions consistent with the Delphi method, and (f) secret balloting of experts in three rounds. Two independent supervisors from abroad oversaw the progression of each stage. The process of preparing recommendations was supported by a methodologist and librarian.

The systematic literature review was carried out independently by 13 people. Pertinent publications were searched for in the following databases: PubMed, OVID, Embase, and Medline. Searches were made using the following Medical Subject Headings: “ultrasonography”, “chest sonography”, “lung ultrasound”, “diagnostic imaging”, “respiratory tract diseases”, “pneumonia”, “pulmonary embolism”, “pneumothorax”, “cardiogenic pulmonary edema”, “non-cardiogenic pulmonary edema”, “lung tumor”, “atelectasis”, “interstitial lung disease”, “pulmonary fibrosis”, “pleural effusion”, “diaphragm”. We excluded such terms as: “endoscopy”, “mammary ultrasonography”, “prenatal ultrasonography”, “endoscopic ultrasound-guided fine needle aspiration”, “intensive care unit”, and “ICU”.

**Figure 1:**
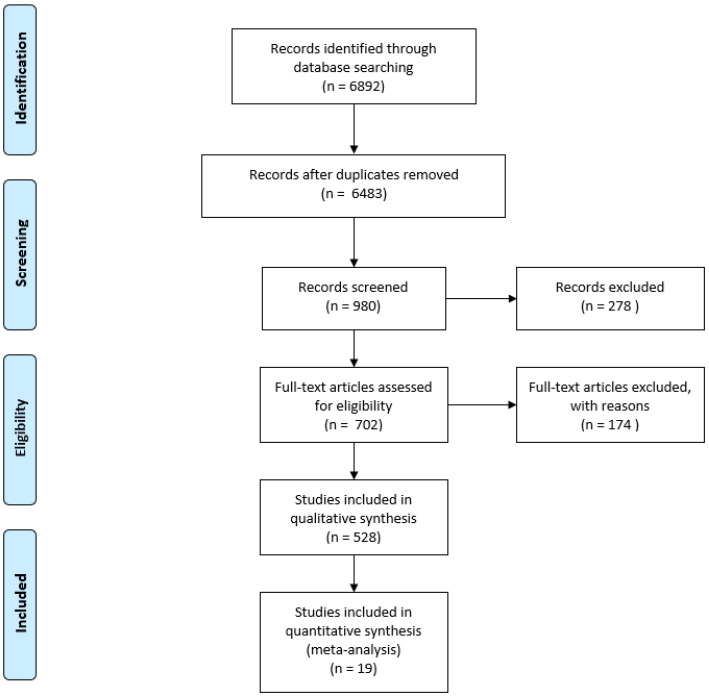
PRISMA flow diagram.

The analysis of literature included prospective, retrospective and observational studies, as well as meta-analyses with their full texts or abstracts published in English prior to November 2019. The exclusion criteria for selecting studies were case reports, reviews (except meta-analyses), letters to the editor and publications related to pediatrics, neonatology, anesthesiology and surgery. The database prepared for the previous version of recommendations was extended with 253 new publications. The initial selection of publications was carried out based on the verification of titles and abstracts, followed by an analysis of the full texts. In the absence of a full text English version, the data provided in the abstract were evaluated. The Zotero program (Center for History and New Media, George Mason University, Fairfax, VA, United States) was utilized to conduct the literature review. The next stage involved the combination of the results of the data search accomplished by the search team, and duplicate papers were removed. Finally, 528 publications were selected for the process of formulating the recommendations, inter alia, 19 meta-analyses, including 6 of those published between September 2017 and November 2019 (Figure 1).

New statements were formulated based on the available data that most frequently appeared in the selected publications. They concerned the following conditions and issues: pneumonia, pneumothorax, pulmonary embolism, heart failure, diagnosis of pleural effusion and of breathlessness, as well as an assessment of hyperhydration in patients receiving dialysis. The established statements first helped in further assessing the literature data’s credibility, which was conducted by a six-person team. Subsequently, the statements were subjected to experts’ opinions.

When analyzing the credibility of the literature data, the following parameters were taken into account: age, sex, number of examined patients, homogeneity of patient groups participating in the study, inclusion and exclusion criteria, publication type (prospective, retrospective, meta-analysis), sensitivity and specificity of the employed method, true positive (TP), false positive (FP), true negative (TN), false negative (FN) results, and the imaging method recognized as the gold diagnostic standard. Moreover, the Tool for the Quality Assessment of Diagnostic Accuracy Studies (QUADAS score and QUADAS score-2), recommended by the Cochrane Diagnostic Test Accuracy Working Group and GRADE (The Grading of Recommendations Assessment, Development and Evaluation), was employed to estimate the methodological quality of the works selected for analysis [2,3,4] (Table 1).

The final opinion formulated by the experts was a result of a three-step procedure conducted via the Internet (online). It consisted of discussions among experts, was carried out according to the modified Delphi method, and involved three rounds of an anonymous ballots featuring the use of personalized passwords. The first round of voting took place on 10–12 January 2020. The statements considered as successfully voted through were those that received ≥ 80% of positive votes. More than 50% of votes against a given statement were considered as unequivocally negating the validity of that statement. Statements that received between 50% and 80% of positive votes were discussed once more and underwent subsequent voting in the second round (Table 2). The second round of voting was held on 16–18 January 2020. In this voting, a consensus was reached with respect to the ambiguous results from the first round. The third and final round of voting was organized on 23–25 January 2020; it concluded with unambiguous results and consensus as to the final recommendations (Table 2 and Table 3).

## 3. Recommendations

### 3.1. Pleural Cavity:

#### 3.1.1. Pneumothorax

1. The sonographic features of pneumothorax are as follows: absence of lung sliding, absence of vertical reverberation artifacts, absence of the lung pulse sign, and the presence of the lung point. (A1)
2. The presence of lung sliding and/or vertical reverberation artifacts rising from the pleural line and/or the lung pulse excludes pneumothorax. (A1)
3. In a patient with acute respiratory failure and with a significant suspicion of pneumothorax, it is not necessary to search for the lung point. (A1)
4. Lung ultrasound is a superior diagnostic imaging technique to chest X-ray for patients with pneumothorax; however, lung ultrasound is less useful than chest X-ray for making therapeutic decisions, such as chest drainage. (A1)
5. Convex and linear transducers are recommended for the diagnosis of pneumothorax. (A1)

Experts’ comments [5,6,7,8,9,10,11,12,13,14,15,16,17,18,19,20,21,22]

(a) The absence of the lung point with the simultaneous presence of pneumothorax occurs in cases of critical or mantle pneumothorax.
(b) Prior pleurodesis affects the presence of lung sliding (the sign will be absent or limited) and of vertical reverberation artifacts (artifacts emerge due to pleural line abnormalities). The presence of vertical artifacts excludes pneumothorax in patients who underwent pleurodesis.
(c) Loculated pneumothorax—a pocket of pleural air can be visualized; the air in the pleural cavity does not move with the change of the patient’s position.
(d) The lung point is the border between the pocket of pleural air and the normal pleural cavity; this sign can be visualized in the B or M-mode.
(e) The lung pulse is the pulse of the lung resulting from cardiac motion transferred to the lung; the lung pulse is best visualized in the M-mode and/or power/color Doppler options.
(f) The recommended position during the examination is the supine position (except for patients with orthopnea).

#### 3.1.2. Pleural Effusion

1. Chest ultrasound is a more sensitive and more specific diagnostic imaging technique for pleural effusion than chest X-ray. (A1)
2. The sensitivity of chest ultrasound, when determining the volume of pleural fluid, is similar to that of chest computed tomography. (B1)
3. Chest ultrasound is a good method for chest imaging that allows for the finding of an optimal place to perform a puncture. (A1)
4. Chest ultrasound allows for the minimization of post-puncture complications. (A1)
5. Sonomorphology of pleural fluid in combination with clinical data may suggest its type. (C1)

Experts’ comments [23,24,25,26,27,28,29,30,31,32,33,34,35,36,37,38,39,40,41]

(a) If available, lung ultrasound should be performed for each patient with a clinical suspicion of pleural effusion and/or in the case when a classical X-ray result indicates the presence of pleural effusion, especially when thoracentesis is required.
(b) Parietal pleural thickening (more than 2 mm) and/or detection of focal lesions within the parietal pleura may suggest a metastatic fluid type.
(c) Ultrasound estimation of the volume of free fluid in the pleural cavity is possible using mathematical formulas. Below are some examples of formulas, dependent on body position:
(d) Sitting position: V (ml) = LH (cm) × 90 or V (ml) = [LH (cm) + SH (cm)] × 70; measurement in posterior axillary line, LH—height of fluid layer, SH—average distance between diaphragm and lung base
(e) Prone position: V (ml) = T (mm) × 20; measurement on exhalation; T—thickness of the fluid layer

### 3.2. LUNGS

#### 3.2.1. Pulmonary Pathologies Associated with Interstitial Pulmonary Lesions

1. The sonographic features of interstitial syndrome are as follows: the presence of lung sliding and ≥3 B-line artifacts in one intercostal space in a single longitudinal scan plane (in relation to the body axis). (A1)
2. Lung ultrasound may be a superior diagnostic strategy to chest X-ray for detecting interstitial lesions. (A1)
3. Interstitial syndrome may be caused by various conditions, including cardiogenic pulmonary edema, non-cardiogenic pulmonary edema, interstitial lung disease, infections and prior bronchoalveolar lavage. (A1)
4. Convex/micro-convex or sector transducers, and, in some cases, a linear transducer, are recommended for the differential diagnosis of the causes of interstitial syndrome. (A1)

Experts’ comments [42,43]

(a) B-line artifacts are laser-like vertical reverberation artifacts arising from the pleural line, extending to the bottom of the screen, and moving along with the movements of the pleural line. The definition of a B-line artifact is based on the use of convex/micro-convex transducers.
(b) The lung sliding sign may be limited or absent in the case of prior pleurodesis or the so-called stiff lung.
(c) A linear transducer is recommended for the differential diagnosis of the causes of “interstitial syndrome”. This is particularly important in the case of bilateral asymmetric interstitial lesions, in the presence of the so-called spared areas, and also in the case of suspected respiratory tract infections, as well as in any clinically ambiguous cause of interstitial pulmonary lesions.

##### Cardiogenic Pulmonary Edema and Heart Failure

1. The sonographic features of cardiogenic pulmonary edema are as follows: most frequently bilateral, gravitational and symmetrical interstitial syndrome, and/or alveolar-interstitial syndrome and/or the white lung sign. (A1)
2. Lung ultrasound is a good diagnostic strategy for diagnosing cardiogenic pulmonary edema. (A1)
3. The use of lung ultrasound in patients diagnosed with heart failure is an important monitoring method during periods of clinical stabilization and exacerbation. (A1)
4. Lung ultrasound performed for patients diagnosed with heart failure allows for the identification of patients at high risk of hospitalization and mortality. (A1)
5. In patients with heart failure, an increased number of B-lines is a predictor of serious cardiovascular events in the near future. (A1)
6. The number of B-lines is a predictor of and correlates with the risk of adverse cardiovascular events, re-hospitalization and mortality for patients with heart failure. (A1)
7. The number of B-lines correlates with an abnormal echocardiogram; hence the detection of B-lines is an indication for performing echocardiography, irrespective of the possible etiology of B-lines. (A1)
8. When monitoring features of pulmonary edema in the context of therapeutic effectiveness, transducers of the same type should be used (convex–convex or sector–sector). (C1)
9. B-line artifacts are a good biomarker that correlate with the level of hyperhydration in patients with heart failure. (B1)
10. In cardiogenic pulmonary edema, the number of B-line artifacts correlates well with the severity of pulmonary edema, NYHA classification (New York Heart Association Classification) and pro-BNP level (pro B-type natriuretic peptide). (A1)
11. An ultrasound image of the lungs of patients with heart failure is useful when deciding on the intensity of diuretic therapy. (A1)
12. In a patient with dyspnea, the absence of B-line artifacts in lung ultrasound excludes the diagnosis of cardiogenic pulmonary edema, and indicates the necessity of searching for other causes of dyspnea. (B1)
13. Lung ultrasound is a good and accurate method differentiating cardiogenic and pulmonary causes of dyspnea. (A1)
14. Lung ultrasound is a superior technique to chest X-ray for imaging cardiogenic pulmonary edema, and is comparable to that of chest computed tomography. (A1)
15. Lung ultrasound is complementary to echocardiography in a clinical assessment of clinically manifested and occult heart failure. (A1)

Experts’ comments [44,45,46,47,48,49,50,51,52,53,54,55,56,57,58,59,60,61,62,63,64,65,66,67,68,69,70,71,72,73,74,75,76,77,78,79,80,81,82,83,84,85,86,87,88,89,90,91,92,93,94,95,96,97,98,99,100,101,102,103,104,105,106,107,108,109,110,111,112,113,114,115,116,117,118]

(a) Interstitial syndrome, alveolar-interstitial syndrome and the white lung sign define successively occurring more advanced stages of interstitial lesions in the course of cardiogenic pulmonary edema. All three of these signs require that at least three B-line artifacts be found in one intercostal space in a single longitudinal scan plane (in relation to the body axis); however, the distance between individual B-line artifacts decreases with an increasing fluid volume in the interstitial space and in the alveoli.
(b) The presence of free-flowing anechoic fluid in pleural cavities may result from heart failure.
(c) The sum of B-lines correlates with the signs of heart failure and the level of natriuretic peptides. It is a predictor of serious cardiovascular events.
(d) Several scales were used in the studies to assess the severity of pulmonary congestion. Examination, consisting of registering 28 scans of the anterolateral chest of a patient in a supine position and summing up the number of registered B-line artifacts, allows for the exclusion of congestion (B-line index <5), or for the revealing of mild (B-line index ≥5 and <15), moderate (B-line index ≥15 and <30) and severe (B-line index ≥30) congestion. This method, although applied in many studies, is, however, time-consuming and requires much experience.
(e) For a quick assessment, the scheme of examining eight or six scans (four or three on each half of the chest, respectively) is suggested—the obtained data may be crucial for rapid diagnostic and therapeutic decisions, but they are qualitative rather than quantitative.
(f) The use of the LuCUS (lung and cardiac ultrasound) protocol (four lung zones on each side of the chest, anterolateral scans, in combination with the assessment of the left ventricular ejection fraction and of the inferior vena cava) is characterized by a sensitivity of 83% and a specificity of 83% in diagnosing acute heart failure as the cause of dyspnea.
(g) A positive correlation between the number of B-line artifacts, clinical signs (NYHA scores) and the level of natriuretic peptides was revealed in the studies. It was revealed that the reduction of the number of B-line artifacts in patients hospitalized due to acute heart failure is positively and linearly correlated with the change in NT-proBNP level (r = 0.44; *p* < 0.05), clinical signs (r = 0.87; *p* < 0.01) and radiologic factors (r = 0.62; *p* < 0.05) [65].
(h) B-lines are defined by a normal pleural line, and are a typical hallmark of cardiogenic pulmonary edema after the exclusion of certain pathologies, including pneumonia or lung contusion, whereas comet-tail artifacts show an irregular pleural line representing a variety of parenchymal lung diseases.

##### Assessment and Monitoring of Dialysis Patients

1. Lung ultrasound is useful for the assessment and monitoring of patients receiving hemodialysis. (C1)
2. B-lines are a good biomarker for the assessment of the degree of pulmonary congestion, and correlate well with other referential methods. (B1)
3. Lung ultrasound is a useful tool for establishing the setting of ultrafiltration volume in dialysis patients. (C1)
4. The number of B-lines in patients receiving hemodialysis increases the risk of hospitalization due to heart failure and the risk of cardiovascular death. (C1)

Experts’ comments [66,119,120,121,122,123,124,125,126,127,128,129,130,131]:

(a) Studies concerning dialysis patients most frequently follow the protocol of assessing 28 scans of the anterolateral chest, adapted from cardiologic examinations. The interpretation of the examination results is sometimes modified by resigning from the notion of “mild congestion”. In such cases, the detection of the sum of B-lines <15 is interpreted as the absence of congestion; terminology applying to other ranges remains unchanged.
(b) Pulmonary congestion revealed in lung ultrasound in patients receiving hemodialysis is significantly, reversely correlated with the quality of life assessed according to the Kidney Disease Quality of Life Short Form (r = −0.22; *p* < 0.001). Pulmonary congestion is an independent factor decreasing the quality of life of these patients, including clinically asymptomatic patients.
(c) Patients with ultrasonographic features of severe congestion, as compared to those with detected features of mild or moderate congestion, present a three-fold greater risk of a cardiovascular event, and a four-fold greater mortality risk (HR = 3.20, 95% CI = 1.75–5.88 for a cardiovascular event and HR = 4.20, 95% CI = 2.45–7.23 for mortality, respectively).

##### Interstitial Lung Disease Involving Pulmonary Fibrosis

1. The sonographic features of interstitial lung disease involving pulmonary fibrosis are as follows: the lung sliding sign, the presence of ≥3 B-line artifacts in one intercostal space (a longitudinal scan plane in relation to the body axis) and pleural line abnormalities. (A1)
2. The use of lung ultrasound may be a superior diagnostic strategy to chest X-ray for diagnosing interstitial lung disease involving pulmonary fibrosis. (A1)
3. The use of lung ultrasound in the monitoring of interstitial lung disease involving pulmonary fibrosis may be helpful (C1)

Experts’ comments [1,42,43,132,133]

(a) Pleural line abnormalities found in patients with pulmonary fibrosis are described as irregular, coarse in appearance, fragmented or blurred.
(b) The use of lung ultrasound in the diagnosis of interstitial pulmonary disease in the active phase is based on case reports, and refers to pulmonary vasculitis, sarcoidosis, hypersensitivity pneumonitis, diffuse alveolar hemorrhage secondary to systemic connective tissue diseases, pulmonary alveolar proteinosis, and interstitial pneumonia secondary to systemic connective tissue diseases.

#### 3.2.2. Pulmonary Pathologies Associated with Consolidations

1. The sonographic features of consolidations are as follows: a subpleural hypoechoic area with a liver-like structure. (A1)
2. The use of lung ultrasound may be a superior diagnostic strategy to chest X-ray for confirming the presence of subpleural consolidations. (A1)
3. Subpleural consolidations may have various underlying causes, most commonly pneumonia, atelectasis (compression- or resorption-related), pulmonary embolism, subpleural neoplastic lesions (primary or metastatic), and lung contusion. (A1)

Experts’ comments [1,134,135]

(a) Experts emphasize the coexistence of multiple morbidities within the respiratory system. The coexistence of more than one respiratory system disease, found in clinical practice, results in the overlapping of several abnormalities in the lung ultrasound scan. It should also be remembered that computed tomography performed according to a protocol suitable for an initial diagnosis is the reference examination in the assessment of pulmonary lesions.

##### Pneumonia

1. The sonographic features of pneumonia are as follows: consolidation, irregular marginal contour, air bronchogram, the air trapping sign, comet-tail artifacts (B-lines), normal vascular pattern in CD and PD (color Doppler and power Doppler) options, and the presence of pleural effusion. (A1)
2. The use of lung ultrasound may be a superior diagnostic strategy to chest X-ray for confirming the presence of pneumonia. (A1)
3. When pneumonia is clinically suspected, the detection of typical inflammatory consolidations in lung ultrasound does not require further confirmation with chest X-ray. (A1)
4. Lung ultrasound is more sensitive and more specific in the diagnosis of community-acquired pneumonia than chest X-ray, and is comparable to the effectiveness of chest computed tomography. (A1)
5. Lung ultrasound is an accurate and quick diagnostic tool for differentiating the causes of acute dyspnea (including pneumonia, acute heart failure and exacerbation of chronic obstructive pulmonary disease (COPD)/asthma). (A1)

Experts’ comments [136,137,138,139,140,141,142,143,144,145,146,147,148,149,150,151,152,153]

(a) Inflammatory lesions are categorized as parenchymatous (consolidation with an irregular marginal contour, dynamic air bronchogram visible within the consolidation and/or the air trapping sign), vascular (normal flow pattern in CD and PD options) and pleural (pleural effusion). This description of lesions does not apply to bronchopneumonia.
(b) Consolidation means an airless area of the lung.
(c) An air bronchogram is the air visible in the bronchial tree within the consolidation.
(d) A dynamic air bronchogram is visible on inspiration and disappears on expiration.
(e) A normal vascular pattern is one that is consistent with the anatomical standard—it is visible with the use of CD and/or PD options.
(f) Experts emphasize that inflammatory lesions caused by tuberculosis, systemic mycosis, pneumocystosis, viral infection and pneumonia of an atypical etiology may present a different sonomorphology than the one described above. It should also be remembered that typical inflammatory lesions may overlap with those caused by less common pathogens.
(g) The diagnostic sensitivity of lung ultrasound for the diagnosis of pneumonia amounts to 87–95%, and specificity to 80–96%. The interpretation of the examination results must account for clinical data. The significance of lung ultrasound results for diagnostic and therapeutic procedures may be particularly important in the following patient groups: geriatric patients, chronically immobile bedridden patients, and patients with chest deformities.

##### Atelectasis

1. The sonographic features of compression atelectasis are as follows: pleural effusion, consolidation of a homogeneous echogenicity and echostructure, static air bronchogram, the air trapping sign, and normal vascular pattern in CD and PD options. (A1)
2. The sonographic features of resorption atelectasis are as follows: consolidation of a homogeneous echogenicity and echostructure, fluid bronchogram, static air bronchogram, normal vascular pattern in CD and PD options, and possible visualization of a pathological mass at the top of the consolidation. (A1)
3. The use of lung ultrasound may be a superior diagnostic strategy to chest X-ray for confirming compression atelectasis. (A1)
4. The use of lung ultrasound may be a superior diagnostic strategy to chest X-ray for confirming resorption atelectasis. (A1)

Experts’ comments [154,155]

(a) Blood flow in CD and PD options is normal only within the area of compression atelectasis, or within the consolidation area constituting resorption atelectasis and not being a pathological mass associated with cancer.
(b) A static air bronchogram represents the presence of air in the bronchial tree and is visible during all respiration phases.

##### Pulmonary Embolism

1. The sonographic features of pulmonary embolism may be as follows: consolidation, mostly wedge-shaped or oval/rounded, centrally located echo, flow amputation in the CD option (the so-called vascular sign), local fluid immediately above the subpleural lesion, and local interstitial lesions. (A1)
2. If pulmonary embolism is suspected, lung ultrasound may be a good diagnostic strategy to confirm the diagnosis. (A1)
3. If pulmonary embolism is suspected (according to the Wells score), the combination of lung ultrasound, transthoracic echocardiography (ECHO) and compression ultrasound (CUS) venous imaging significantly increases the sensitivity and specificity of pulmonary embolism diagnosis, and consequently allows for limiting the number of angio-CT exams performed. (A1)
4. Pulmonary embolism is diagnosed if ≥ 2 typical abnormalities are found in lung ultrasound. (B1)
5. The diagnosis of pulmonary embolism is likely if one abnormality typical of pulmonary embolism is detected in lung ultrasound. (C1)
6. The absence of abnormalities typical of pulmonary embolism does not rule out its diagnosis. (A1)

Experts’ comments [156,157,158,159,160,161,162,163,164,165,166]

(a) In the case of patients with acute respiratory failure and a high risk of death, it is recommended to follow the BLUE protocol and criteria for the diagnosis of pulmonary embolism designed therein as a potential cause of the patient’s acute status.
(b) Lung ultrasound may be an alternative technique for the diagnosis of pulmonary embolism when angio-CT cannot be performed or is contraindicated, e.g., in pregnant women, patients with acute kidney failure, and patients allergic to contrast agents.
(c) The use of lung ultrasound in the diagnosis of pulmonary embolism may increase the sensitivity and specificity of commonly applied assessment scores for the clinical risk of embolism (e.g., the Wells score); it does not, however, apply to the assessment of the patient’s prognosis.
(d) Two thirds of the lung infarctions are located dorsally in the lower lobes of the lungs. Therefore, it is very important to examine the patients from the dorsal in the right- and left-side position.
(e) In about 50% of the cases there are small pleural effusions, focally above the lesions in the pleural cavity.

##### Subpleural Malignant Lesions

1. The sonographic features of subpleural malignant lesions are as follows: infiltration of adjacent structures, diverse sonomorphology of the consolidation, chaotic vascular pattern in CD and PD options, and concomitant resorption atelectasis and/or fluid. (A1)
2. The use of lung ultrasound is a good diagnostic strategy during invasive procedures (transthoracic biopsy) in the diagnosis of subpleural lesions suspected of being malignant. (A1)
3. Lung ultrasound is a good imaging technique that allows for the localization of peripheral pulmonary tumors adjacent to the chest wall, or tumors within the consolidation area below the fluid in the pleural cavity. (B1)

Experts’ comments [1,167,168]

(a) Subpleural malignant lesions may be accompanied by accessory vascularization originating from intercostal vessels that can be visualized in CD and PD options.
(b) The use of ultrasound guidance during a biopsy is applicable for both subpleural lesions and biopsies through the acoustic window formed by fluid or atelectasis.

### 3.3. Diaphragm 

The ultrasound assessment of diaphragm function in patients with chronic obstructive pulmonary disease (COPD) may prove useful when predicting therapeutic effectiveness (e.g., non-invasive ventilation (NIV)). (C1)

Experts’ comment [169,170,171,172,173,174]

Ultrasonography can assess the characteristics of diaphragmatic movement, such as amplitude, force and velocity of contraction, special patterns of motion, and changes in diaphragmatic thickness during inspiration.

### 3.4. Other Indications

1. The use of lung ultrasound may be a good diagnostic strategy for determining the causes of dyspnea. (A1)
2. The use of lung ultrasound may be a good diagnostic strategy for the differential diagnosis of pleuritic chest pain. (A1)
3. The use of lung ultrasound may be a good screening strategy for the differential diagnosis of acute cough (A1)
4. A lung ultrasound examination performed by a trained clinician is at a comparable level to a lung ultrasound examination performed by a radiologist. (A1)

Experts’ comments [1,120,158,175,176]

**Figure 2:**
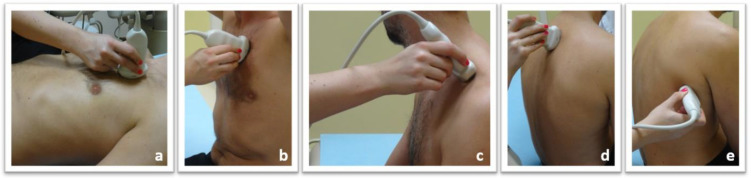
Lung ultrasound technique for internal medicine patients: Lung ultrasound examination in patients with internal diseases is performed with the convex and linear probe, in the supine and/or sitting position: (**a**) Scanning the anterior chest wall in the supine position; (**b**) Scanning the anterior chest wall while seated; (**c**) Assessment of the peaks through the supraclavicular fossa; (**d**) Assessment of the posterior chest wall in a sitting position; (**e**) Assessment of the posterolateral thoracic wall in a sitting position.

Publications clearly indicate that a bedside lung ultrasound examination performed by trained clinicians is a better solution than transporting the patient to a radiology unit to perform lung ultrasound there. The clinician has data from the patient’s medical history and physical examination, as well as knowledge as to the patient’s present status, which all impact on the accuracy of the final diagnosis. The technique of performing a lung ultrasound examination for internal medicine patients is presented in Figure 2.

## 4. Experts’ Additional Suggestions

1. Bedside examination is recommended for a clinically unstable patient with dyspnea.
2. The lung ultrasound scanning technique depends on the patient’s clinical status and should cover the largest possible area of the lungs.
3. Lung ultrasound performed by a trained clinician for a patient with respiratory failure is a good and safe diagnostic constituent for the differential diagnosis of pulmonary diseases.
4. Basic training in the theoretical aspects and practical use of lung ultrasound is recommended for physicians during their specialty training programs, including internal medicine, cardiology, pulmonology and nephrology.
5. The recommended basic course for clinicians during their specialty training programs should cover the diagnosis of pleural effusion, pneumothorax, cardiogenic and non-cardiogenic pulmonary edema, interstitial lung disease involving fibrosis, pneumonia, atelectasis, pulmonary embolism, subpleural malignant lesions, rib fracture as well as assistance during invasive diagnostic procedures and therapies.
6. It is recommended to incorporate basic lung ultrasound training into the curriculum of students at faculties of medicine in medical universities.

Experts’ comment

Convex (or possibly micro-convex or sector) and linear transducers are recommended when examining a patient in a stable clinical condition. The patient can be examined in a sitting position or a lying position (except for patients in a forced position or with orthopnea, for whom the examination is performed in a sitting or semi-sitting position).

## 5. Practical Aspects of Lung Ultrasound Examination

There are many proposed protocols for lung ultrasound in the literature. Some are dedicated to the quick assessment of a patient with acute dyspnea (e.g., BLUE protocol), while others are used in the semi-quantitative assessment of pulmonary congestion in patients with heart failure (protocol 28 scans, also used in haemodialysis patients, or its modifications). The authors of this document propose to follow the following general principles of examination:

1. When examining the patient, the protocol appropriate to the clinical situation should be selected each time;
2. If the patient’s condition allows it, assess the anterior, lateral and posterior surfaces of the chest in the vertical and supine positions of the patient, using a convex probe and then a linear probe (Figure 2);
3. When analyzing the obtained ultrasound image, first of all, answer the following questions:
  (a) Was the pleural line visible on the entire lung fields during the examination?
  (b) is lung sliding present?
  (c) is the pleural line correct?
  (d) are there any pathological artifacts?
  (e) if pathological artifacts are found, their location should be determined (according to the anatomical topography of the chest)
  (f) if any subpleural consolidations are found, provide their location, dimensions, shape, echogenicity, and the degree of separation from the environment. If technically possible, an assessment of the vascularization (doppler) is also advisable.
  (g) are the pleural cavities free of fluid?
  (h) if fluid is found in the pleural cavity, its echogenicity should be determined and its volume should be estimated
4. In everyday clinical practice, the results of the ultrasound examination of the lungs should be interpreted similarly to the results of other additional examinations, i.e., in relation to the overall clinical picture.

**Figure 3:**
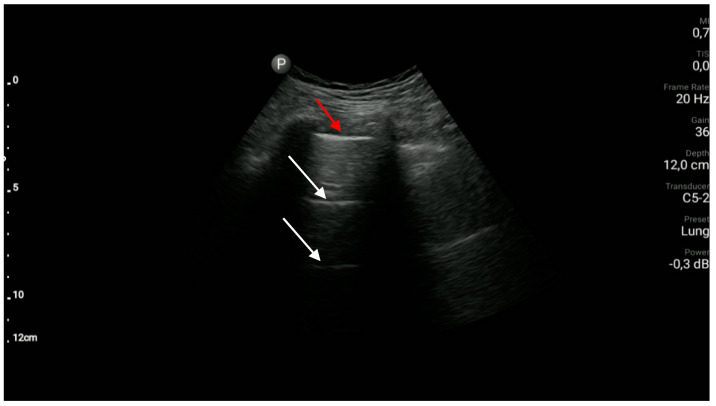
Normal pleural line (red arrow) and A-line artefacts (white arrows).

**Figure 4:**
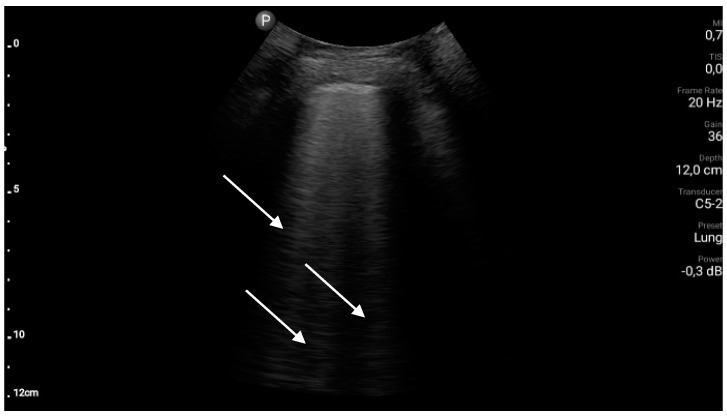
B-line artefacts (white arrows).

**Figure 5:**
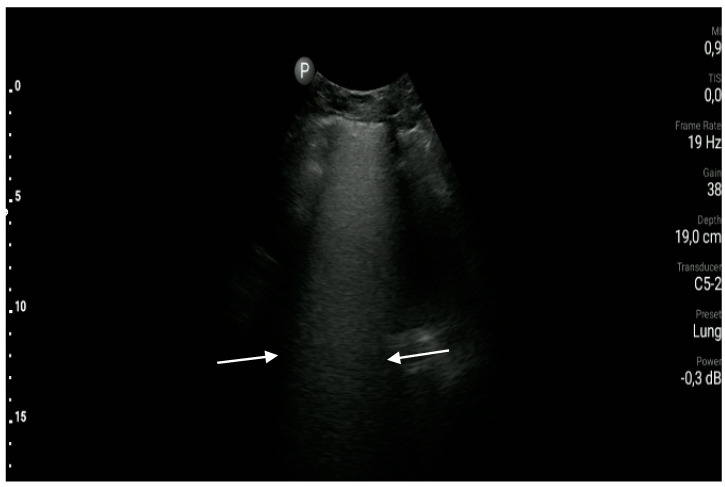
So called “white lung”, a significant number of B-lines which cannot be visualized separately (area between white arrows).

**Figure 6:**
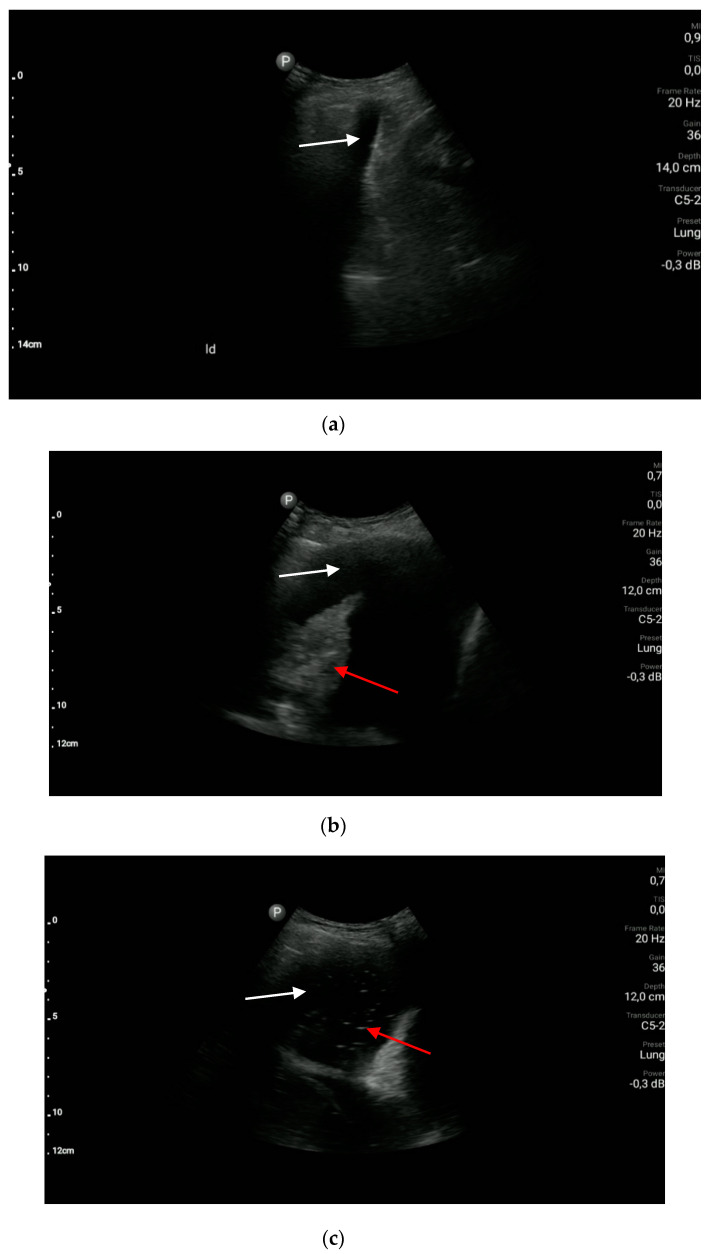
Pleural effusion: (**a**) a small amount of anechoic fluid (white arrow); (**b**) a massive amount of anechoic fluid (white arrow) and atelectasis (red arrow); (**c**) a massive amount of fluid (white arrow) and hyperechoic inclusions (red arrow) suggesting pleural empyema.

**Figure 7:**
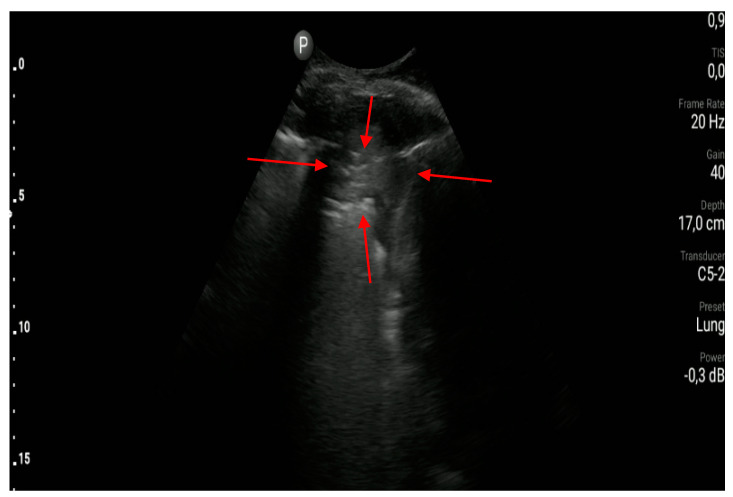
Subpleural consolidation (between red arrow).

Exemplary ultrasound images along with a brief description are shown in Figure 3, Figure 4, Figure 5, Figure 6 and Figure 7.

## 6. Conclusions

Large amounts of data from scientific publications confirm our belief that lung ultrasound as a lung imaging technique is presently an important diagnostic tool in the everyday clinical practice of specialists in internal medicine and related specialties. The experiences of clinicians gathered during the last two years has provided new data allowing for the reaching of a consensus related to diagnostic issues in, among others, patients with heart failure and pneumonia, and patients receiving dialysis. Lung ultrasound facilitates a rapid and efficient diagnosis or suspicion of specific pulmonary diseases. Consequently, the utilization of lung ultrasound expedites decisions regarding the introduction of an appropriate therapy, or the extension of the diagnostic process as compared to the classical diagnostic procedures. These recommendations will be updated along with the accumulation of new, reliable reports in medical literature.

## Figures and Tables

**Table 1:** Degree of experts’ agreement, Delphi method.

Voting Results	Conclusion	Designation or Reaction
≥80% positive votes	Agreed for	1
≤50% positive votes	Agreed against	2
51–79% positive votes	Indeterminate	Statement re-debated and voted on in second and/or the third round voting

**Table 2:** Quality of evidence, GRADE.

Grade	Interpretation
A	High—data from multiple meta-analyses, and/or it is unlikely that further research will change the credibility of the effectiveness or accuracy of the method
B	Moderate—data from individual large non-randomized trials (meta-analysis, prospective cohort study), and/or further testing may have a significant impact on the credibility of the effectiveness or accuracy of the method
C	Low or very low—agreed expert opinion and/or data from small studies, retrospective studies, registers, case series, or case reports, and/or it is very likely that further testing will have an important impact on the credibility of effectiveness or accuracy of the method. Any estimation of the effects or accuracy of the method is very uncertain (very low)

**Table 3:** Strength of recommendations and its practical implications.

Strength of Recommendation	Interpretation and Practical Implication of Recommendation
1A	strong recommendation; the given procedure should be widely used, as long as there are no strong contraindications
1B	strong recommendation, but with a smaller degree of certainty; probably right in most individual cases
1C	the average (mean) strength of recommendation; the recommendation may change after obtaining more reliable data; probably right
2A	the average (mean) strength of recommendation; the decision on its adoption is a matter of choice and may depend on local and individual conditions; intervention does not have to be used
2B	weak recommendation; alternative conduct can be just as good or better
2C	weak recommendation; alternative treatment is probably equally acceptable
